# The complexity of invasive fungal diseases in the intensive care unit: evaluation of metagenomic next-generation sequencing

**DOI:** 10.3389/fcimb.2026.1820501

**Published:** 2026-05-29

**Authors:** Rui Wang, Hailing Yang, Chunmei Zhang, Xirenguli Zi Neng

**Affiliations:** 1 Department of Geriatrics, China-Japan Union Hospital of Jilin University, Changchun, Jilin, China; 2 Department of Emergency Medicine, China-Japan Union Hospital of Jilin University, Jilin, Changchun, China; 3 Department of Critical Medicine, China-Japan Union Hospital of Jilin University, Jilin, Changchun, China; 4 Department of Critical Medicine, The Sixth Affiliated Hospital of Xinjiang Medical University, Xinjiang, Ürümqi, China

**Keywords:** diagnostic efficacy, immune state, intensive care unit, invasive fungal diseases, metagenomic next-generation sequencing

## Abstract

**Background:**

In the intensive care unit (ICU), a subset of adult individuals who are non-neutropenic and lack conventional host risk factors frequently develop fungal infections, which constitute a major mortality risk in this population. This patient group has received limited attention to date, and research on diagnostic approaches remains insufficient. This research looks into whether metagenomic next-generation sequencing (mNGS) could be used to diagnose this group of people.

**Methods:**

We performed a retrospective analysis of 106 individuals with invasive fungal infections between July 2022 and February 2025. These patients were divided into two groups: immunocompetent and immunocompromised. Demographic and clinical characteristics were analyzed and compared between the two groups. The diagnostic value of mNGS was carefully assessed, and its diagnostic performance was contrasted with that of conventional microbiological tests (CMTs). In addition, the impact of mNGS results from different specimen types on clinical management and antifungal treatment decisions was summarized.

**Results:**

Among the 106 adult patients, 66.26% were immunocompetent, but many of them had underlying comorbidities. A total of 81 pathogens were identified, of which 74 were detected by mNGS and 44 by CMTs. The predominant fungal pathogens included *Candida species*, *Pneumocystis jirovecii*, and *Aspergillus fumigatus*. mNGS showed a distinct superiority in identifying uncommon pathogens and mixed infections, with its total positive rate markedly exceeding that of CMTs. mNGS results led to beneficial modifications in clinical management for 75 patients (70.75%). The clinical impact varied by specimen type, including bronchoalveolar lavage fluid (BALF; 61 cases), blood (14 cases), and other sterile body fluids (31 cases), with blood specimens yielding the least clinical benefit.

**Conclusion:**

In the ICU, a substantial number of invasive fungal infections occur among patients without classical host risk factors. mNGS offers substantial benefits in identifying fungal pathogens and mixed infections, hence enhancing the diagnostic efficacy of invasive fungal diseases (IFDs). The extent of clinical benefit is affected by the kind of specimen provided for testing.

## 1 Introduction

Invasive fungal diseases (IFDs) are well-known to be frequent among people with weak immune systems, such as those with neutropenia or who have had a solid organ transplant. They are also one of the main causes of death in these people. It is now known, however, that certain adult patients who are brought to the intensive care unit (ICU) and do not have neutropenia or the other host characteristics often have fungal infections (Muskett et al., 2011; Bassetti and Bouza, 2017; Ostrosky-Zeichner and Al-Obaidi, 2017; Bao et al., 2022). The management of IFDs is time-sensitive; any delay in diagnosing the disease or starting the right antifungal treatment raises the risk of death. Among all diagnostic methods, microbial culture continues to play a crucial role. It is still the most widely used method for detecting bacteria and fungi and is considered the foundation of diagnosis. However, the sensitivity of the cultures varies according to the kind of IFDs. The sensitivity for candidemia may reach 80%; for other deep-seated Candida infections, it may be as low as 21% (Ostrosky-Zeichner and Al-Obaidi, 2017). Moreover, some microbial species have stringent nutritional requirements and cannot be readily cultured, resulting in an even lower detection rate. Currently, a variety of non-culture detection methods have been adopted in clinical practice to expedite the diagnostic process, including serological assays and molecular biological techniques (Jia et al., 2023a). Among molecular biological techniques, pan-fungal polymerase chain reactions (PCRs) have advanced rapidly, enabling the direct identification of fungal pathogens. These molecular tools comprise pan-fungal PCRs targeting multicopy ribosomal deoxyribonucleic acid (DNA) regions (ITS1/ITS2, 18S, 28S rDNA), as well as targeted PCR assays for genera such as *Aspergillus, Candida, Mucorales, Pneumocystis jirovecii, Cryptococcus neoformans*, and locally prevalent fungi (e.g., *Sporothrix*). These PCR-based methods make it possible to directly find many different fungal pathogens in clinical samples without the need for prior culture, thereby significantly shortening turnaround time (Donnelly et al., 2020; Jenks et al., 2023; Schelenz et al., 2025). Nonetheless, these approaches target only a limited spectrum of pathogens and consequently cannot fully meet the demands of clinical diagnosis.

Metagenomic next-generation sequencing (mNGS) is a groundbreaking technique that is unbiased and doesn’t rely on any assumptions. Its main benefit is that it can detect uncommon and hard-to-cultivate pathogens. Compared with traditional culture-based methods, it provides a more comprehensive view of the microbial community (Tan et al., 2024; Zhao et al., 2024). mNGS has been preliminarily used to diagnose and optimize therapeutic regimens for a variety of infectious diseases, including central nervous system (Wang et al., 2023b; Benoit et al., 2024), respiratory system (Hao et al., 2023; Gao et al., 2024), hematologic system (Liu et al., 2021; Li et al., 2023), gastrointestinal system (Nie et al., 2023; Guo et al., 2024), urinary system (Jia et al., 2023b; Wang et al., 2023c), prosthetic joints (Wang et al., 2023a), and skin and soft tissues (Liu et al., 2024), among others. Nevertheless, mNGS still has some limitations, as the optimal specimen types and sampling timing for detection remain unclear, alongside a labor-intensive and high-cost experimental workflow, and it also relies on high-performance computational infrastructure to complete subsequent data analysis (Elbehiry and Abalkhail, 2025; Schelenz et al., 2025). Despite these constraints, mNGS is especially well suited for critically sick patients with complicated diseases and quickly progressing illness because of its excellent accuracy and quick turnaround. mNGS features extensive pathogen coverage and an untargeted, hypothesis-independent detection mode. Because patients in the ICU often have complicated illnesses and a large range of infection kinds and locations, mNGS has significant diagnostic value (Xi et al., 2022; Liang et al., 2023). However, the majority of mNGS investigations have focused on bacterial infections, with relatively few studies addressing fungal infections, which have mostly been explored among immunocompromised populations. The community of fungal infections in ICU wards is exceedingly varied, exhibiting a diverse array of baseline comorbidities while lacking the typical host characteristics associated with IFDs (Bassetti et al., 2024). Fungal infections in this population have received insufficient attention, and studies on diagnostic methods are very limited. To address this gap, our research evaluated the diagnostic efficacy of traditional approaches vs. mNGS in ICU individuals with IFDs. We assessed the effect of mNGS on antimicrobial therapy in immunocompetent and immunocompromised individuals and reported how mNGS findings from various specimen types influenced treatment choices.

## 2 Materials and methods

### 2.1 Study design and participants

**Figure 1:**
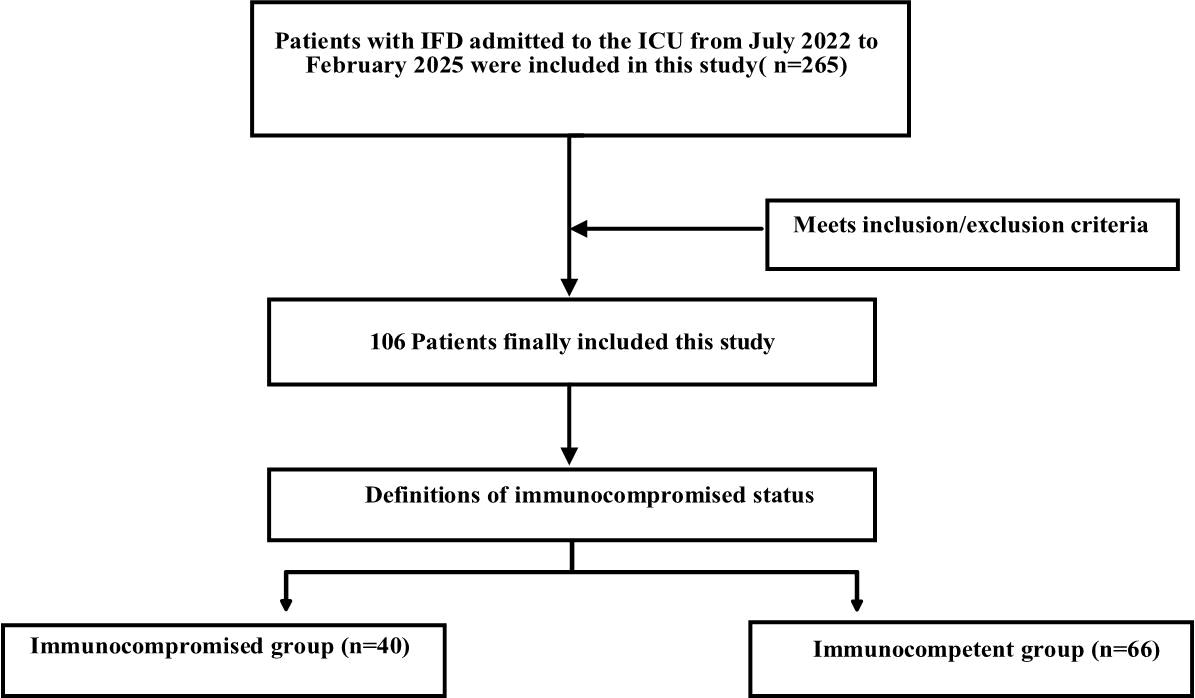
Study the flowchart. Initially, 265 patients with IFDs were screened. Based on the inclusion criteria set by the study, a total of 106 patients were enrolled and analyzed according to traditional host factor criteria, dividing them into the immunocompetent group and the immunocompromised group.

This retrospective research included 265 patients clinically diagnosed with IFD, who were hospitalized in the ICU of the China–Japan Union Hospital from July 2022 to February 2025. According to the established screening criteria, 106 individuals were finally included in the research (**Figure 1**).

The criteria for inclusion were as follows: (1) adults aged 18 and over; (2) a preliminary diagnosis of IFD with antifungal therapy administered during hospitalization; (3) conventional microbiological tests (CMTs) and mNGS were submitted for testing at the same time; (4) identical specimen types were used for both mNGS and CMTs.

The criteria for exclusion were as follows: (1) refusing to participate in mNGS testing; (2) specimen leakage or contamination, or insufficient clinical records; (3) inability to meet mNGS quality control standards; (4) uncertain prognosis during hospitalization.

### 2.2 Collection of clinical specimens

Various specimen types were collected based on the presumed infection location. The specimen types were blood, pus, BALF, drainage fluid, pleural effusion, and ascitic fluid. Bronchoalveolar lavage fluid (BALF) was procured using electronic bronchoscopy; peripheral blood samples were gathered in instances with unexplained fever or where the infection site was challenging to identify; additional specimens were obtained via sterile puncture or drainage. Upon collection, all specimens were transported to the laboratory within one hour, according to their specific storage protocols, and concurrently submitted for mNGS analysis.

### 2.3 Conventional microbiological tests

This study adopted CMTs for pathogen detection, covering two major components: microbial culture and nucleic acid PCR assay. The culture-based assays consisted of blood culture, BALF culture, and other bodily fluid cultures. For blood culture, 8–10 mL of peripheral blood was injected into Anaerobic/F Culture Vials and Aerobic/F Culture Vials, followed by incubation on the BACTEC FX system for 7 days, with extended incubation for up to 14 days for fungal isolation. Cultivation was kept in a 5% CO_2_ incubator at 35 °C and a routine incubator at 28 °C. China blue and blood agar plates were quantitatively inoculated with urine samples. Other body fluid specimens were directly streaked onto blood agar, China blue agar, and Sabouraud agar, followed by incubation at 35 °C and 28 °C in a standard incubator, with microbial growth monitored daily. PCR detection was only used to find viral pathogens, including *influenza A/B, coronavirus, respiratory syncytial virus, rhinovirus, adenovirus*, and *Mycoplasma pneumoniae*. Serological tests include the galactomannan (GM) test and the serum (1, 3)-β-D-glucan (BDG) assay. Imaging examination, smear microscopy (excluding special staining), and serological tests were collectively used to assist clinical diagnosis and therapeutic efficacy evaluation.

### 2.4 mNGS procedures

#### 2.4.1 Sample preparation and genetic material extraction

This study included many types of clinical samples for pathogen detection. Negative controls were simultaneously established throughout the experiment to effectively eliminate interference from exogenous contamination. Different pretreatment strategies were used for different sample types and characteristics. Routine urine was processed by centrifugal enrichment and resuspension, while hematuria samples received host DNA removal. Conventional drainage fluid samples were treated with standard procedures, whereas host removal was performed for bloody drainage fluid. Pleural effusion, pus and ascites were uniformly processed for host DNA removal regardless of blood content. Peripheral blood samples were separated into plasma by centrifugation, and no additional treatment was required for bloody blood specimens. Routine BALF was processed directly before library construction, while bloody BALF received host depletion treatment.

Peripheral venous blood (5 mL) was collected using BCT tubes and centrifuged at 1600 g for 10 minutes, with plasma harvested for cell-free DNA extraction. The plasma supernatant was harvested for cell-free DNA extraction. For BALF, 5 mL specimens were fully mixed. Viscous samples were diluted with Genekey sample diluent (Cat. No. 2008) at a 1:1 volume ratio to reduce viscosity. After dilution, the proportion of human-derived nucleic acids was quantified. Specimens with a high human nucleic acid proportion were treated with host depletion reagents, centrifugal enrichment, constant-temperature incubation, and repeated precipitation and centrifugation to reduce interference from human host sequences.

DNA from experimental samples and negative batch controls was extracted with the Genekey Micro DNA Kit (1901), with nucleic acid concentration and purity assessed via the Qubit dsDNA HS Assay Kit. Genomic DNA of patient specimens and negative controls was extracted in parallel using the TIANamp Micro DNA Extraction Kit (DP316, TIANGEN). Total ribonucleic acid (RNA) was isolated with the Genekey RNA Extraction Kit (2005-01), and RNA concentration was quantified by Qubit detection reagents and a fluorescence detector. DNA extraction from host-depleted lavage fluid samples was conducted meticulously following the manufacturer’s guidelines using the Genekey magnetic bead-based nucleic acid extraction kit (2005-01). After uniform pre-treatment, consistent DNA and RNA extraction procedures were implemented for all samples to ensure experimental homogeneity.

A bead-beating lysis method was employed to disrupt microbial pathogens efficiently. Lysis parameters were set at 18 m/s for 420 seconds in the standard workflow and 18 m/s for 140 seconds for host-depleted procedures. These lysis parameters were determined exclusively by experimental workflows rather than sample types. An RNase-free, sterile environment was maintained throughout the whole experiment, with staged disinfection using ethanol, hydrogen peroxide, and ultraviolet irradiation performed before, during, and after testing to strictly control cross-contamination.

#### 2.4.2 Library construction and sequencing

For the development of the library, nucleic acid samples that passed quality control were used. An NGS library preparation kit with unique dual index (UDI) adapters was used to create partial DNA libraries, which were then purified using 0.6× AMPure XP magnetic beads and rinsed using 20 μL of 1 M Tris-HCl buffer. The Genekey NGS Library Construction Kit (2012B) was used to create the remaining DNA libraries. DNA extracted from lavage fluid and other specimens was processed by the dedicated Genekey pathogen UDI library construction kit (2012), purified with 0.9× purification magnetic beads, and finally eluted and recovered using 32 μL elution buffer.

Using the Genekey reverse transcription kit (2053A), cDNA was initially created for RNA samples before libraries were built. The Agilent 2100 Bioanalyzer was used to evaluate library quality, and the Qubit 1× dsDNA HS Assay Kit and Qubit 4.0 detector were used to estimate concentration. Selected libraries were precisely quantified by qPCR. The qualified concentration threshold was no less than 1 nmol/L for DNA libraries and above 1 M for RNA libraries, with full compliance with PIM maximum data volume standards.

This work used standardized high-throughput sequencing parameters. Sequencing was conducted on the MGI-200 platform with the SE50 + 10 + 10 mode. All samples shared identical sequencing depth with only differentiated data volume requirements. In routine workflows, the raw data volume threshold was no less than 20 M for clinical DNA samples and 5 M for RNA samples, while negative controls required more than 10 M raw data for DNA and 5 M for RNA. For host-depleted workflows, both clinical samples and negative controls reached a minimum DNA data volume of 5 M, and RNA detection was not conducted. The overall sequencing quality control criteria included a Q30 ratio ≥85% and GC content <45%.

The Illumina NextSeq 550 and MGISEQ-200 platforms were used to sequence qualified libraries. All host-depleted bloody BALF samples were uniformly sequenced on the MGI-200P platform using a single-end 50 bp strategy. Multiple negative controls were included in each experimental batch. Internal reference markers were added to the DNA workflow to monitor cross-contamination, with no positive or blank controls set for the RNA detection system.

#### 2.4.3 Raw data quality control and host sequence filtering

Raw BCL data produced by sequencing were transformed into FASTQ format with Bcl2fastq software. Quality checking and filtering of raw reads were conducted using fastp, KZ, and Komplexity programs to eliminate adapter contamination, low-quality bases, and low-complexity reads, resulting in high-quality clean data. The Bowtie2 tool was used to align clean reads against a human reference genome database dominated by the hg38 assembly and supplemented with non-human primer sequences. Mapped host sequences were discarded, and valid microbial reads were retained for subsequent taxonomic annotation and analysis.

#### 2.4.4 Result interpretation and quality control screening

Filtered valid microbial sequences were aligned to a non-redundant database covering bacteria, fungi, viruses, and parasites using SNAP software for taxonomic annotation. The analysis consisted of initial pathogen genome alignment and definitive species classification. The lowest common ancestor algorithm was applied for accurate taxonomic assignment when single reads matched multiple closely related species. Samtools and Bedtools were used to calculate pathogen genome coverage and average sequencing depth as indicators of result reliability. Species with valid reads fewer than 3 were verified by secondary alignment against the NT database to avoid false-negative results.

The pathogen reference database was constructed based on the Manual of Clinical Microbiology, Diagnosis and Interpretation of Clinical Microbiology, as well as the NCBI RefSeq and GenBank databases, with one high-quality representative strain selected per species. The current detection system covers 9, 855 bacteria, 6, 926 viruses, 1, 582 fungi, 312 parasites, 177 mycobacteria, and 184 mycoplasma/chlamydia species.

Strict filtering was applied to preliminary analytical results to exclude abnormal alignments with genome coverage < 1% and sequencing depth > 2, as well as background contaminating flora. Background contaminants were defined as environmental and reagent-derived bacteria with read counts within the laboratory’s normal historical range relative to negative controls. Pathogen analysis and positive judgment were performed in combination with control results. The top 10 most abundant bacterial, viral, and parasitic species were screened separately. Suspicious pathogens were comprehensively validated by integrating clinical symptoms, imaging findings, and laboratory indicators to ensure scientific and credible reports.

All bioinformatic analyses were performed in triplicate to guarantee result stability and repeatability. Statistical analysis was conducted using SPSS software. A total of 259 digital reference samples across three categories (closely related strains, simulated clinical specimens, and rare pathogen models) were applied to fully validate the performance of the analytical workflow. Data analysis was completed based on Python, which realized unified calculation of classification efficiency indicators, statistical analysis, and visualized plotting, so as to comprehensively ensure the stability and accuracy of the overall bioinformatics analysis pipeline.

### 2.5 Clinical evaluation

IFDs denote a severe infectious ailment characterized by the invasion of fungi into deep human tissues or sterile bodily cavities. The presence of fungal elements must be confirmed via direct microscopic examination, histopathology, or culture from sterile sites, accompanied by tissue damage or inflammatory responses. Invasive Candidiasis occurs when Candida species invade normally sterile sites including the bloodstream, abdominal cavity, liver, kidneys, and spleen, triggering candidemia or deep tissue infection, accompanied by infectious symptoms, inflammatory reactions, or functional impairment of affected organs. Invasive Aspergillosis is defined by the invasion of *Aspergillus* into the lung parenchyma, airway, and paranasal sinuses, or hematogenous dissemination to other organs, causing tissue necrosis, invasive inflammation, and corresponding clinical infectious manifestations. Pneumocystis jirovecii Pneumonia is an infectious interstitial pneumonia resulting from the invasion of *Pneumocystis jirovecii* in the alveoli and lung interstitium, clinically presenting as cough, dyspnea, and hypoxemia, characterized by bilateral ground-glass opacities on imaging (Hage et al., 2019; Donnelly et al., 2020; Bassetti et al., 2021, Bassetti et al., 2024). These definitions differentiate IFDs from simple fungal colonization, contamination, or non-invasive parasitism on mucosal or cutaneous surfaces. Notably, the detection of fungal components through direct microscopic examination of specimens collected from sterile sites is sufficient for a definitive IFD diagnosis (Schelenz et al., 2025).

Clinical diagnosis: Two specialists independently assessed the existence of a fungal infection and its location. During the process, they evaluated the patient’s clinical history, immune status, clinical symptoms and signs, imaging findings, mNGS read abundance, culture results, laboratory test outcomes including serological tests and PCR, together with the efficacy of antifungal treatment. Utilizing the combined data, the experts assessed whether the patient had IFDs and provided a final clinical diagnosis. Disagreements were addressed through additional discussions; if consensus remained elusive, a multidisciplinary consultation of laboratory specialists, radiologists, senior doctors, and bioinformatics experts was organized. The definitive clinical diagnosis was obtained in accordance with pertinent criteria and expert consensus (Donnelly et al., 2020; Bassetti et al., 2021, Bassetti et al., 2024).

A patient is defined as immunocompromised if any of the listed risk factors are present.

1. Hematologic malignancies.
2. History of allogeneic stem cell or solid organ transplantation, or grade III or IV acute graft-versus-host disease.
3. Recent neutropenia (<0.5 × 10^9^ neutrophils/L or <500 neutrophils/mm³) persisting for more than 10 days.
4. Prolonged corticosteroid therapy (excluding patients with allergic bronchopulmonary aspergillosis) at a dose of ≥ 0.3 mg/kg for ≥ 3 weeks within the past 60 days.
5. Receipt of immunosuppressive therapy within the past 90 days.
6. Primary or secondary immunodeficiency disorders.
7. End-stage hepatic or renal disease.

### 2.6 Statistical analysis

Data processing was accomplished using SPSS 31.0 software. The differences between groups for categorical data were analyzed using the χ² test. For measurement data, after normality testing, non-normally distributed data were described as median (interquartile range) M (P25, P75), and the comparison between groups was conducted using the Mann-Whitney U test. Normally distributed data were described as mean ± standard deviation ( $\bar{\text{x}}$ ± s), and the comparison between groups was conducted using the t-test. The significance level was set at α = 0.05, and p < 0.05 was considered statistically significant. GraphPad Prism 10.0 was used to draw histograms, pie charts, and ring charts. The distribution and proportion of diseases in the normal group and immunocompromised group were analyzed, the consistency of different detection methods was compared, and the mNGS detection results of different specimen types were evaluated.

## 3 Results

### 3.1 Patient characteristics and distribution of patients

**Figure 2:**
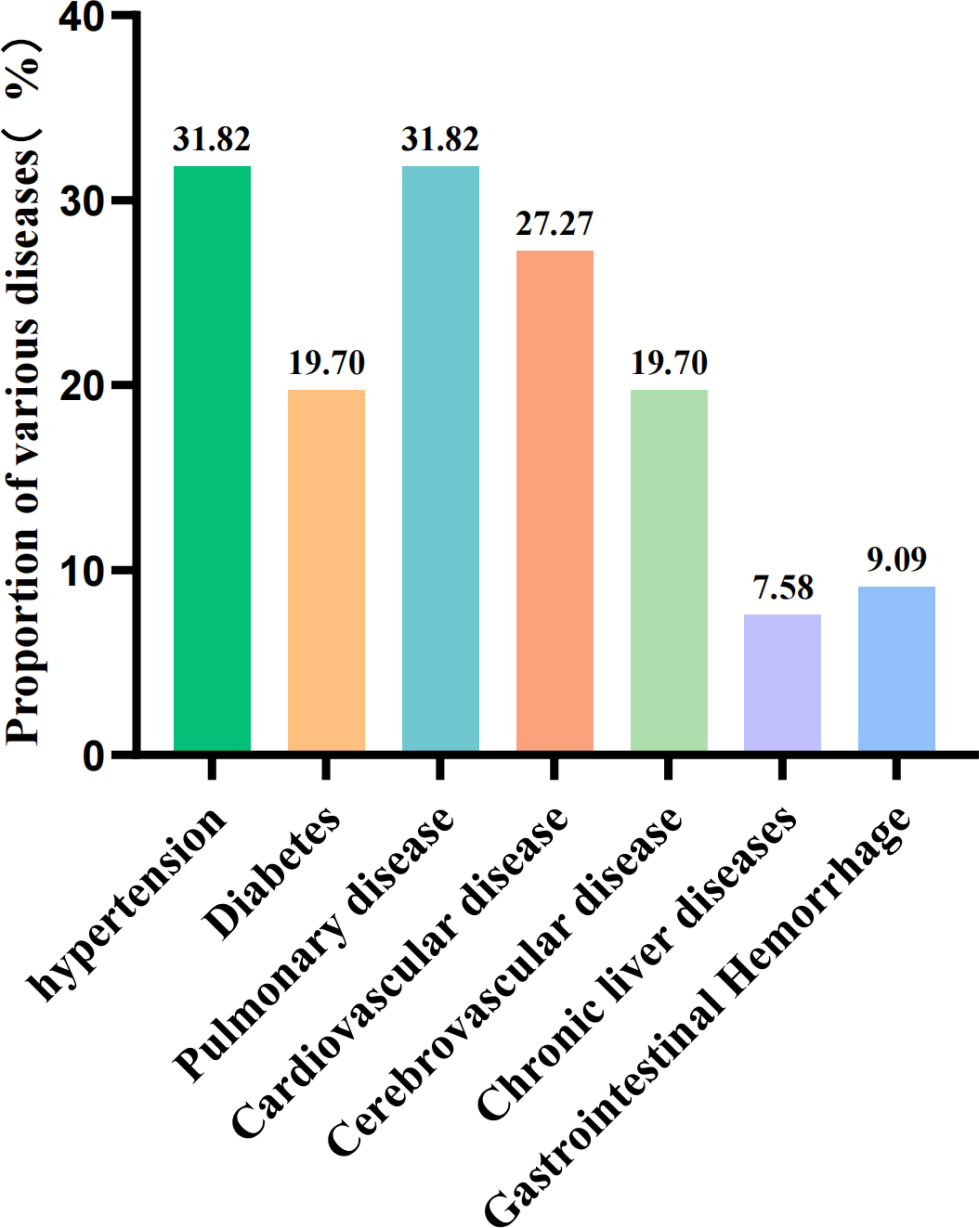
The distribution of underlying diseases among the 66 patients in the immunocompetent group.

**Figure 3:**
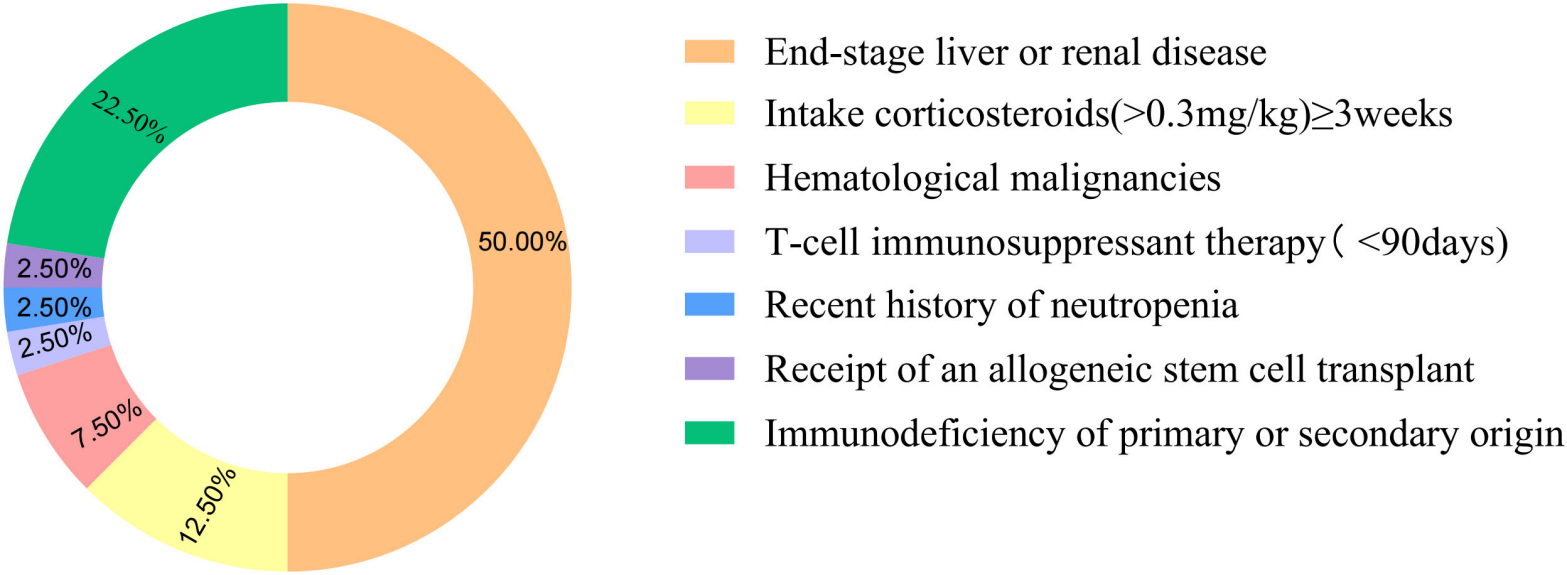
Distribution of causes affecting immune function in the group of 40 immunocompromised patients.

This research encompassed 106 patients. Of the total, 67 were male and 39 were female. All patients received a clinical diagnosis of invasive fungal infections. Patients were classified into two groups based on their immune function status: an immunocompetent group (n = 66) and an immunocompromised group (n = 40). Among the 66 patients in the immunocompetent group, 31.82% had hypertension, 31.82% had respiratory diseases, 27.27% had cardiovascular diseases, 19.70% had cerebrovascular diseases, 19.70% had diabetes mellitus, 9.09% experienced gastrointestinal bleeding, and 7.58% had chronic liver disease. Thirty patients had two or more comorbid underlying diseases, accounting for 45.45% of the group (**Figure 2**). Among the 40 immunocompromised patients, 50.00% had end-stage liver or renal disease; 22.50% had primary or secondary immunodeficiency; 12.50% had received corticosteroid therapy (dose >0.3 mg/kg for ≥3 weeks); and 7.50% had hematological malignancies. In addition, 2.50% of patients had received T-cell immunosuppressant therapy within the previous 90 days, had a recent history of neutropenia, or had received allogeneic hematopoietic stem cell transplantation (**Figure 3**).

We analyzed the basic characteristics of the patients, including basic information such as gender and age; clinical feature of fever; comorbidities including hypertension, diabetes, pulmonary disease, cardiovascular disease, cerebrovascular disease, chronic liver diseases, and surgical site infection; clinical laboratory data including white blood cell count (WBC), neutrophils count, lymphocyte count, C-reactive protein (CRP), procalcitonin (PCT), albumin (ALB), D-dimer, GM, BDG; and ICU treatment including mechanical ventilation, frequent hospitalizations, ICU mortality, and symptom improvement. Regarding basic information, the immunocompromised group was slightly younger than the immunocompetent group. The clinical symptom of fever differed significantly between the two groups (*P* < 0.05) and was more prevalent in immunocompromised patients. In terms of comorbidities, hypertension was the most common and showed a significant difference between the two groups (*P* < 0.05), with a higher prevalence in the immunocompromised group compared to the immunocompetent group. Additionally, it is noteworthy that the incidence of postoperative infections was higher, and all cases were observed in the immunocompromised group. In clinical laboratory data, PCT and D-dimer showed significant differences between the two groups (*P* < 0.05), with levels significantly elevated in the immunocompromised group. No significant differences were observed for the remaining indicators. For ICU treatment, no differences were found between the two groups for any of the parameters (**Table 1**).

**Table 1:** Comparison of baseline characteristics between the two groups.

Characteristic	Total (n=108)	Immunocompetent (n=66)	Immunocompromised (n=40)	*P* value
Age(M, IQR)( $\bar{x}$ ± *s*)	70 (57, 76)	71 (59, 76)	70 ± 3.34	0.036*
Male(n, %)	67(63.21%)	43(65.20%)	24(60.00%)	0.594
Female(n, %)	39(36.79%)	23(34.80%)	16(40.00%)	
Clinical features				
Fever(n, %)	78(73.58%)	44(66.67%)	34(85.00%)	0.038*
Comorbidity				
hypertension(n, %)	42(39.62%)	21(31.82%)	21(52.50%)	0.035*
Diabetes(n, %)	24(22.64%)	13(19.70%)	11(27.50%)	0.352
Pulmonary disease(n, %)	37(34.91%)	21(31.82%)	16(40.00%)	0.392
Cardiovascular disease(n, %)	29(27.36%)	18(27.27%)	11(27.50%)	0.980
Cerebrovascular disease(n, %)	21(19.81%)	13(19.70%)	8(20.00%)	0.970
Chronic liver diseases(n, %)	12(11.32%)	5(7.58%)	7(17.50%)	0.118
Surgical Site Infection(n, %)	14(13.21%)	0(0.00%)	14(35.00%)	<0.001**
Clinical laboratory data				
WBC, ×10^9^/L(M, IQR)	12.47 ± 1.25	10.88 ± 1.07	22.96 ± 4.32	0.182
neutrophils, ×10^9^/L(M, IQR)	9.52(6.15, 14.63)	8.50(6.08, 13.97)	21.25 ± 4.39	0.195
lymphocyte, ×10^9^/L(M, IQR)	0.61(0.43, 0.94)	0.61(0.42, 0.92)	1.00 ± 0.22	0.494
CRP, mg/L(M, IQR)( $\bar{x}$ ± *s*)	130.19 ± 15.91	134.08 ± 17.47	104.55 ± 38.82	0.630
PCT, ng/mL(M, IQR)	0.77(0.34, 7.36)	0.53(0.19, 4.07)	3.00(0.70, 13.80)	0.003*
ALB, g/L(X̄±σ)	29.29 ± 0.79	28.75 ± 0.85	32.80 ± 1.30	0.196
D-dimer, µg/mL(M, IQR)	4.75 (1.64, 11.58)	3.70(1.43, 8.82)	14.32 ± 4.51	0.029*
GM, µg/L(M, IQR)	0.26 (0.25, 0.39)	0.26(0.25, 0.35)	0.25(0.25, 0.39)	0.814
BDG, pg/mL(M, IQR)	66.64 (37.5, 192.75)	67.16(37.50, 185.36)	54.29(37.50, 194.63)	0.953
ICU treatment				
Mechanical Ventilation(n, %)	87(82.08%)	55(83.33%)	32(80.00%)	0.665
Frequent Hospitalizations(n, %)	17(16.04%)	11(16.67%)	6(15.00%)	0.821
ICU Mortality(n, %)	24(22.64%)	17(25.75%)	7(17.50%)	0.325
Symptom Improvement (n, %)	76(71.70%)	35(53.03%)	21(52.50%)	0.958

*P<0.05, **P < 0.001.

### 3.2 Analytical performance of mNGS in contrast with CMTs

We evaluated the diagnostic consistency between mNGS and CMTs. All 106 enrolled patients underwent both mNGS and CMTs testing. The final definitive diagnosis was determined by comprehensive clinical evaluation. Kappa analysis was performed accordingly. The Kappa value was 0.016, less than 0.4, indicating poor agreement between mNGS and CMTs. In addition, the positive detection rate of mNGS was significantly higher than that of CMTs (**Table 2**).

**Table 2:** Kappa analysis of concordance between mNGS and CMTs results.

mNGS	CMTs		Total
	(+)	(-)	
(+)	43	55	98
(-)	3	5	8
Total	46	60	106

*P=0.77*, Kappa value=0.016.

**Figure 4:**
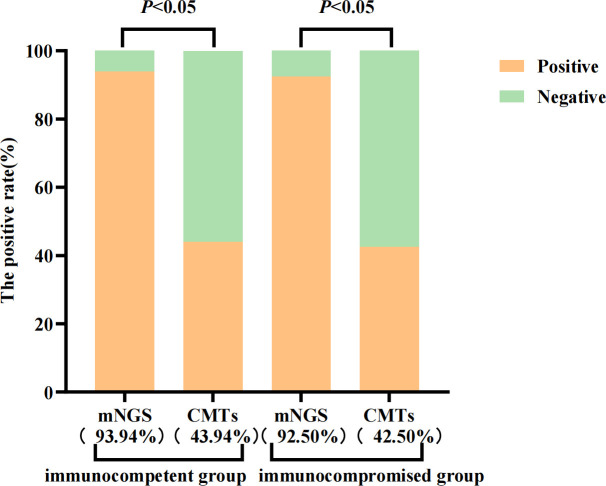
Comparison of positive detection rates between mNGS and CMTs.

In the grouped patient population, the positive rate of mNGS was much superior to that of CMTs in both the immunocompetent and immunocompromised groups (93.94% vs 43.94%, *P* < 0.05; 92.50% vs 42.50%, *P* < 0.05). No significant variations were seen in mNGS or CMT positive rates between groups (**Figure 4**).

**Figure 5:**
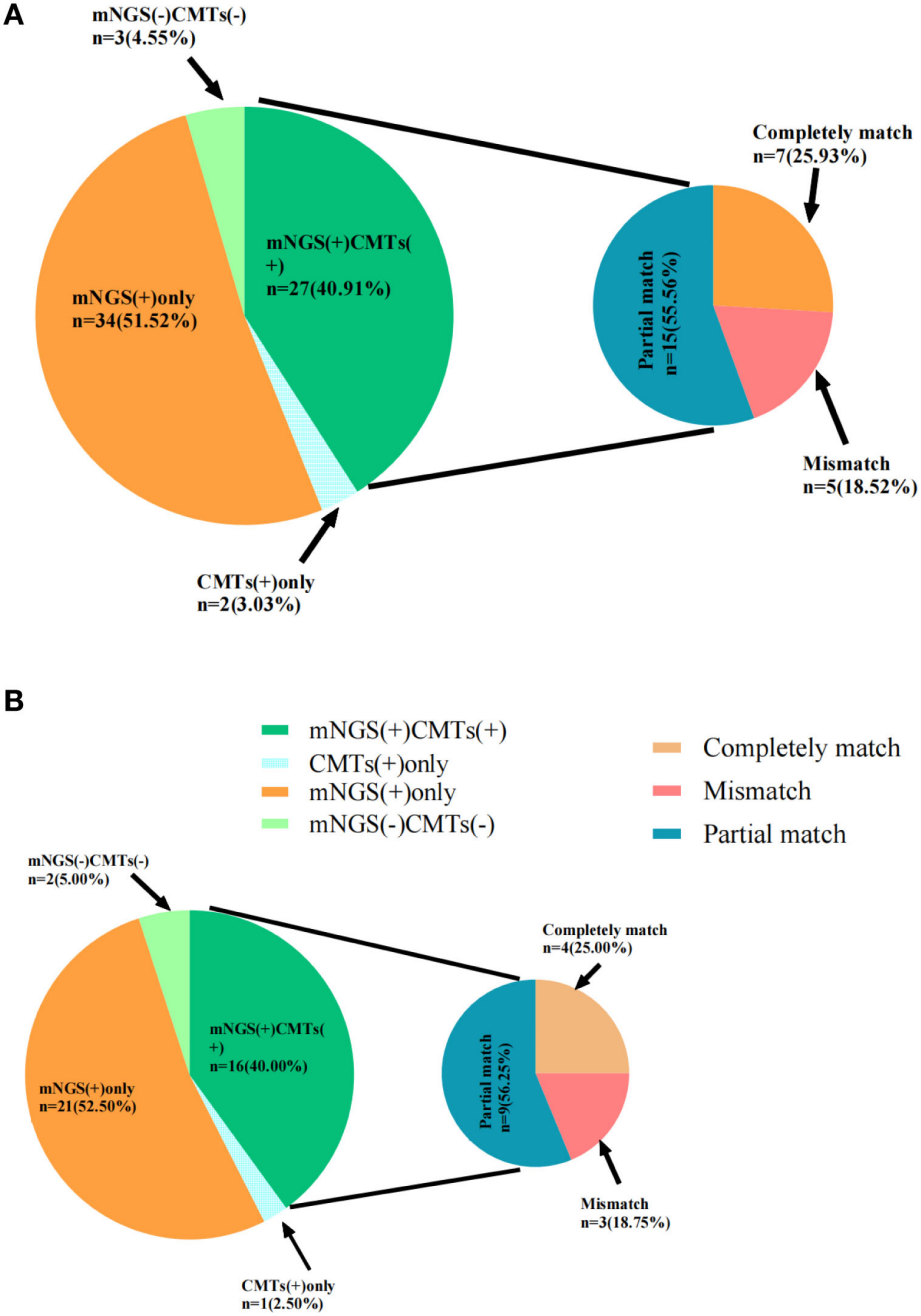
The consistency between metagenomic sequencing and traditional culture methods in pathogen identification. **(A)** The immunocompetent group. **(B)** The immunocompromised group.

Among the 66 immunocompetent patients, 2 cases (2/66, 3.03%) were positive only by CMTs, whereas 34 cases (34/66, 51.52%) were positive only by mNGS. Three cases (3/66, 4.55%) were negative by both methods, and 27 cases (27/66, 40.91%) were positive by both methods. Among these 27 double-positive cases, complete concordance between the two methods was observed in 7 cases (7/27, 25.93%), partial concordance in 15 cases (15/27, 55.56%), and complete discordance in 5 cases (5/27, 18.52%) (**Figure 5A**). Among the 40 immunocompromised patients, 1 case (1/40, 2.50%) was positive only by CMTs, whereas 21 cases (21/40, 52.50%) were positive only by mNGS. Two cases (2/40, 5.00%) were negative by both methods, and 16 cases (16/40, 40.00%) were positive by both methods. Among these 16 double-positive cases, complete concordance between the two methods was observed in 4 cases (4/16, 25.00%), partial concordance in 9 cases (9/16, 56.25%), and complete discordance in 3 cases (3/16, 18.75%) (**Figure 5B**).

### 3.3 Variations in the detection of pathogenic microorganisms by CMTs and mNGS

**Figure 6:**
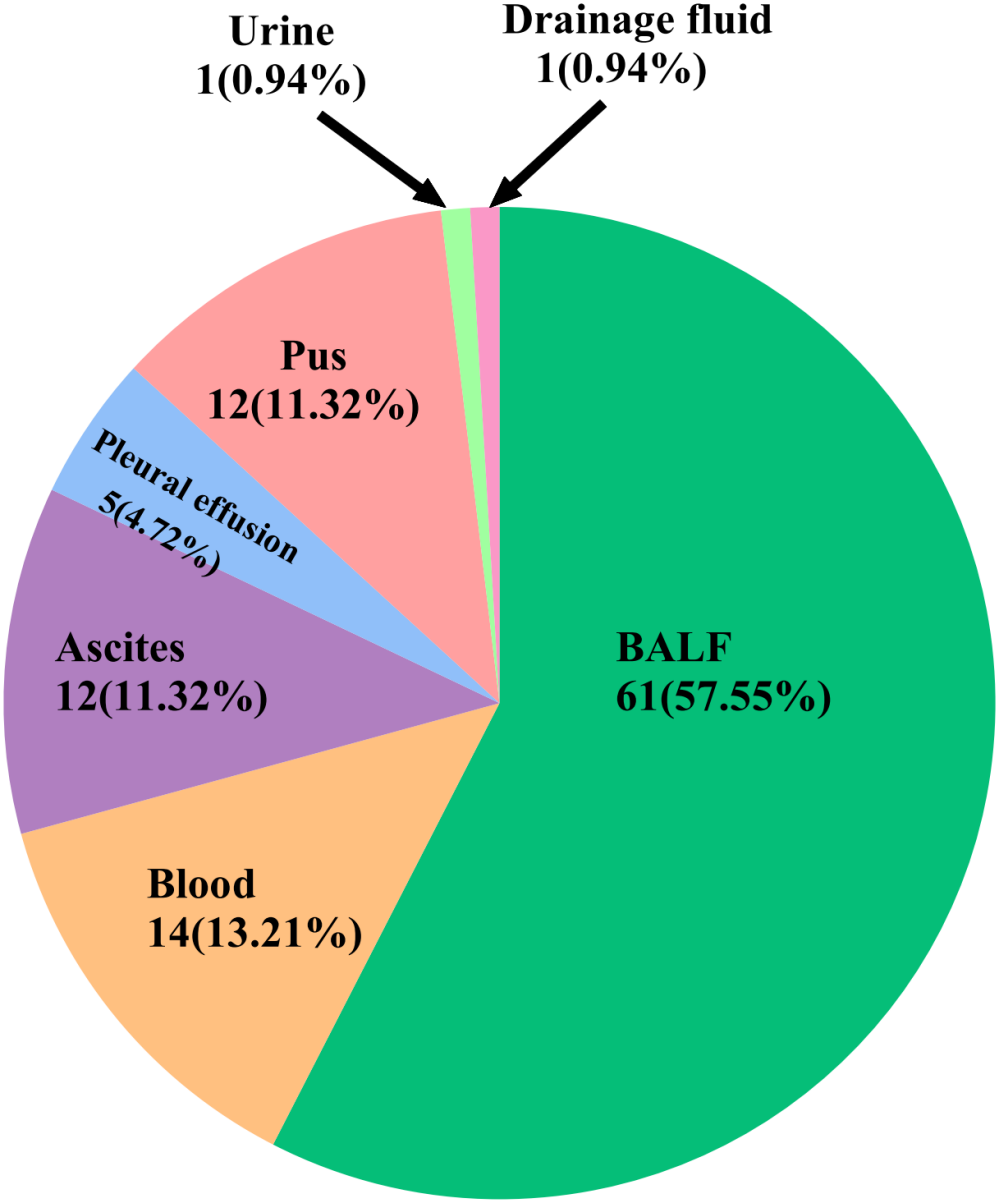
Proportional distribution of specimen types among all samples submitted for testing.

**Figure 7:**
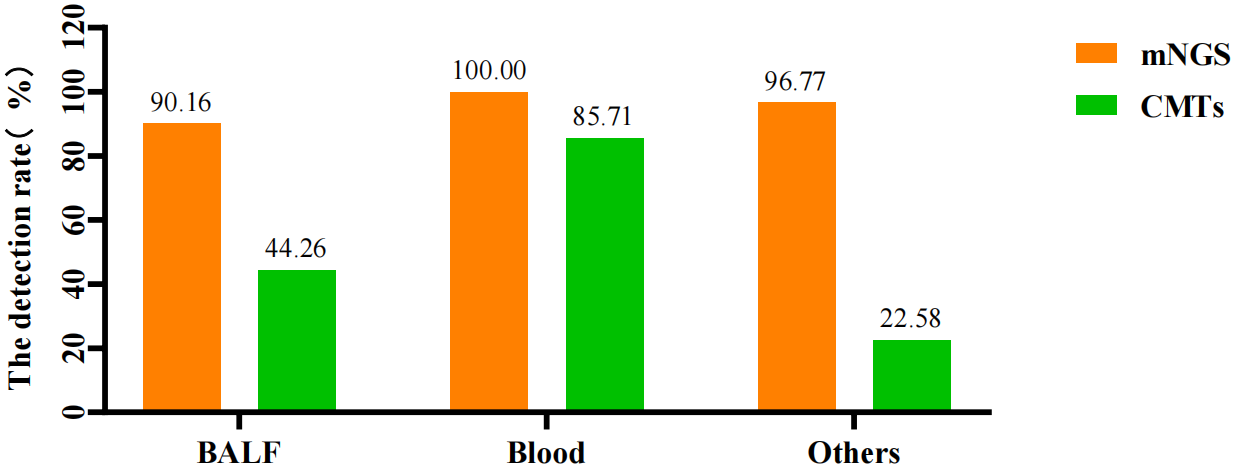
Comparison of pathogen-positive detection rates between mNGS and CMTs across different specimen types. The “others” group included pus, pleural effusion, urine, drainage fluid, and ascitic fluid.

In this study, a total of seven types of clinical specimens were included. BALF accounted for the largest proportion (57.55%), followed by blood samples (13.21%), ascitic fluid (11.32%), pus (11.32%), pleural effusion (4.72%), urine (0.94%), and drainage fluid (0.94%) (**Figure 6**). The fungal detection rate of mNGS was substantially greater than that of CMTs in BALF and sterile body fluid specimens (including urine, drainage fluid, ascitic fluid, pleural fluid, pus, and pleural effusion) (*P* < 0.05). Interestingly, nevertheless, there was no discernible difference in the fungus detection rate in blood samples between mNGS and CMTs (100.00% vs. 85.71%, *P* > 0.05) (**Figure 7**).

**Figure 8:**
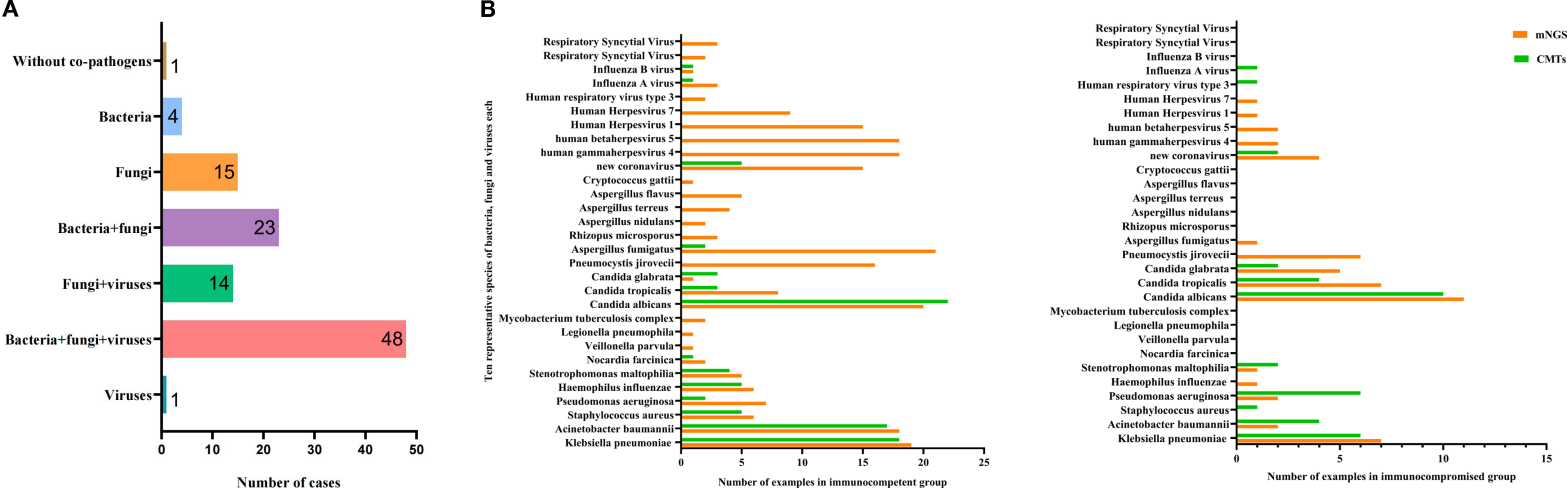
mNGS pathogen detection results. **(A)** Types of fungal co-infections, with the bar chart showing the number of cases for each type of mixed infection. **(B)** Comparison of detection counts of representative bacteria, fungi and viruses by mNGS and CMTs in immunocompetent and immunocompromised groups.

Comparison of Bacterial Detection: In the immunocompetent group, mNGS and CMTs showed similar detection numbers for *Klebsiella pneumoniae, Acinetobacter baumannii, Staphylococcus aureus*, and *Stenotrophomonas maltophilia.* mNGS detected more *Pseudomonas aeruginosa, Haemophilus influenzae, Nocardia farcinica, Veillonella parvula, Legionella pneumophila*, and *Mycobacterium tuberculosis*, which were rarely or not detected by CMTs. In the immunocompromised group, CMTs yielded slightly higher detection counts for *Pseudomonas aeruginosa, Acinetobacter baumannii*, and *Stenotrophomonas maltophilia* than mNGS. mNGS was superior in detecting *Haemophilus influenzae*; CMTs failed to identify other rare bacteria such as *Nocardia, Legionella*, and *Mycobacterium tuberculosis*. The two methods were comparable for common bacteria, whereas only mNGS could detect rare, fastidious, and non-culturable bacteria. Comparison of Fungal Detection: In the immunocompetent group, detection of *Candida albicans* was similar between mNGS (20) and CMTs (22). Detection rates of *Candida tropicalis* and *Candida glabrata* were significantly higher with mNGS than with CMTs. mNGS successfully identified numerous cases of *Pneumocystis jirovecii, Aspergillus fumigatus, Rhizopus microsporus, Aspergillus nidulans, Aspergillus terreus, Aspergillus flavus*, and *Cryptococcus gattii*, while CMTs detected almost none of these pathogens. In the immunocompromised group, detection of *Candida albicans* was comparable between the two methods. mNGS detected more *Candida tropicalis* and *Candida glabrata*. *Pneumocystis jirovecii* and *Aspergillus fumigatus* were detected by mNGS but not by CMTs; CMTs failed to detect other molds. Fungal detection represents the core advantage of mNGS, especially for *Pneumocystis jirovecii* and Aspergillus species, which are nearly undetectable by conventional methods. Comparison of Viral Detection: In the immunocompetent group, mNGS identified *coronavirus*, *herpesviruses types 4, 5, 1*, and *7*, *influenza A/B, respiratory syncytial virus*, and *torque teno virus 16*. CMTs only detected a small number of *coronavirus, influenza A*, and *influenza B* cases, with no detection of any *herpesviruses, respiratory syncytial virus*, or *torque teno virus.* In the immunocompromised group, mNGS detected *coronavirus* and *herpesviruses types 4, 5, 1*, and *7*. CMTs only identified a few cases of *coronavirus, human respiratory virus type 3*, and *influenza A*, with no detection of *herpesviruses*. CMTs had almost no capacity for viral detection, while mNGS could simultaneously identify (**Figure 8B**).

### 3.4 Impact on the management of infections

**Figure 9:**
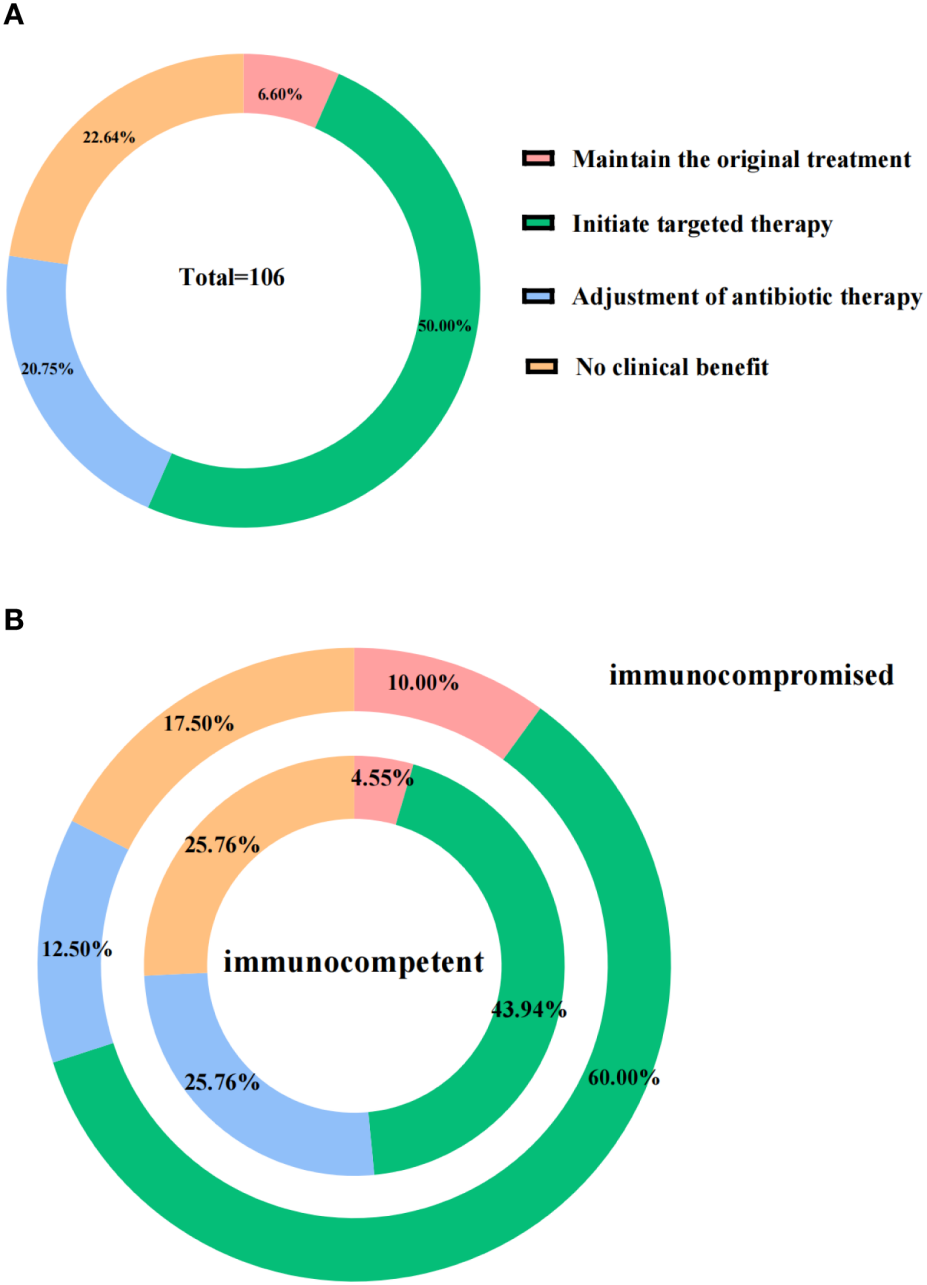
Impact of mNGS results on anti-infective therapy, with 75 cases showing a beneficial effect. **(A)** Influence of mNGS results on clinical treatment plans in 106 patients. **(B)** Evaluation of clinical treatment outcomes in immunocompromised patients (outer ring) and immunocompetent patients (inner ring). The proportion of patients receiving clinically beneficial therapy was similar between the two groups.

mNGS positively influenced the therapy of 75 patients (70.75%). The clinical benefits included initiating targeted therapy in 53 patients (53/106, 50.00%) and adjusting antibiotic therapy in 22 patients (22/106, 20.75%) (**Figure 9A**). The proportion of clinically beneficial treatments based on mNGS results was similar between the immunocompromised and immunocompetent groups (72.50% vs. 69.70%). However, compared to the immunocompetent group, the impact on the immunocompromised group was more focused on initiating targeted therapy (60.00% vs. 43.94%) (**Figure 9B**).

In the immunocompromised population, the rates of adjunctive antifungal and antiviral therapy initiation were both notably higher in the mNGS group (*P* < 0.05). No statistically significant difference was observed regarding simple antifungal regimen adjustments between the two groups. Additionally, mNGS-guided treatment was associated with a significantly lower ICU mortality rate (*P* < 0.05).In the immunocompetent group, mNGS guidance was associated with significantly higher rates of initiation of antifungal therapy, initiation of antiviral therapy, and antifungal regimen adjustment compared to CMTs. Additionally, mNGS-guided treatment resulted in a significantly lower ICU mortality rate than CMTs, with all differences being statistically significant (**Table 3**).

**Table 3:** The impact of antibiotic regimen adjustments and patient prognosis based on mNGS and CMT results.

Clinical impact	Immunocompetent group(n=66)		Immunocompromised group(n=40)	
	mNGS	CMTs	mNGS	CMTs
Adjunctive antifungal	28(42.42%)*	18(27.27%)	24(60.00%)*	13(32.50%)
Adjunctive antiviral	17(25.76%)**	4(6.06%)	14(35.00%)*	7(17.50%)
Adjustment of antifungal treatment	14(21.21%)	4(6.06%)	5(12.50%)*	1(2.50%)
ICU Mortality	7(11.11%)*	10(34.48%)	2(5.56%)*	5(35.71%)

Comparison the antibiotic regimen adjustments and patient prognosis between guided by mNGS and CMTs in immunocompromised group or immunocompetent group. *P < 0.05, **P < 0.001.

Regarding different specimen types, the clinical benefit of initiating targeted therapy based on mNGS results was highest in ascitic fluid (66.67%) and pleural effusion (80.00%), followed by BALF (47.54%). The impact of adjusting antibiotic therapy based on mNGS results was most notable in pus (25.00%) and BALF (21.31%), followed by ascitic fluid (8.33%). For blood specimens, the majority of patients continued their original treatment regimen (78.57%), yielding the lowest clinical benefit. There was 1 urine specimen, which led to the initiation of targeted therapy, and 1 drainage fluid specimen, which resulted in adjustment of the antibiotic regimen (**Supplementary Material**).

## 4 Discussion

We evaluated the occurrence of IFDs among immunocompetent and immunocompromised patients in the ICU and compared the diagnostic efficiency of mNGS and CMTs for IFDs. We observed a particular group of ICU patients who lacked established host susceptibility factors and were immunocompetent; their post-infection clinical and laboratory findings were largely comparable to those of immunocompromised patients, with no significant difference in mortality. This population deserves focused attention. We also found that mNGS had a wider detection spectrum and higher sensitivity than CMTs in immunocompetent and immunocompromised patients. For common bacteria, the two methods were comparable, but mNGS was superior in identifying rare, fastidious, and non−culturable pathogens. Fungal detection represented the core advantage of mNGS, especially for *Pneumocystis jirovecii* and molds that were rarely detected by CMTs. For viruses, mNGS identified a broad range of pathogens, whereas CMTs missed most viruses, particularly *Herpesviruses*. Overall, mNGS effectively identifies mixed and occult infections, providing reliable evidence for precise anti−infective therapy, timely regimen adjustment, and better prognosis in critically ill patients (Lv et al., 2023). In addition, appropriate selection of specimen types for testing is equally important in supporting accurate pathogen identification and optimization of therapeutic strategies.

According to the latest estimates, the annual incidence of invasive fungal infections is approximately 6.5 million, with 3.8 million deaths worldwide, of which about 2.5 million can be directly attributed to fungal infections (Denning, 2024). Most work on invasive mycoses centers around immunocompromised patients. However, recent reports have shown that the severely ill adult patients in the ICU who do not have neutropenia are heterogeneous, including both internal medical and surgical cases. Patients have different co-morbidities with several risk factors, which make them prone to IFDs (Azim and Ahmed, 2024; Bassetti et al., 2024; Martin-Loeches, 2024). This research further substantiates the occurrence of invasive fungal infections in immunocompetent patients in the ICU. Immunocompetent patients comprised 62.26% of the included cases, surpassing those with immunocompromised conditions. This discovery indicates that the patient group with invasive fungal infections in the ICU has unique characteristics and lacks the commonly recognized host risk factors, highlighting the necessity of enhancing clinical vigilance. Moreover, end-stage liver disease and renal disease were mostly seen in the immunocompromised group, contrasting with results from earlier research (Wang et al., 2022), which may be ascribed to disparities in patient characteristics across various clinical departments. The analysis of the population structure in this research indicated that patients in the immunocompromised group were marginally younger, and the incidence of fever exhibited a significant difference between the two groups when compared to immunocompetent patients. Aside from PCT and D-dimer, no substantial variations were seen in other laboratory parameters, suggesting that baseline clinical symptoms and routine examination results were mostly similar across the two groups. This result indicates that in the immunocompetent group, due to the absence of well-established host factors, clinical fungal infections tend to be more insidious. Furthermore, in immunocompetent populations, the reduced incidence of fever may lead to an underestimation of infections in terms of early clinical diagnosis. One more noteworthy result: All instances of postoperative fungal infection were seen only in the immunocompromised cohort. Prior studies indicate that postoperative fungal infections significantly elevate the risk of short-term mortality (Wei et al., 2025), underscoring the need for enhanced postoperative infection monitoring in this demographic.

The diagnosis of invasive fungal infections generally relies on the identification of the etiological agent. Fungal culture is laborious and time-consuming, necessitating spore development and subsequent macroscopic and microscopic identification, frequently resulting in delayed etiological knowledge (Schwarz et al., 2024). Previous studies have shown that mNGS can swiftly provide extensive pathogen profiles and enable prompt targeted treatment (Han et al., 2019). Furthermore, mNGS is essential for detecting fungal infections in patients with negative CMTs, thereby enhancing treatment outcomes (Shi et al., 2023). This research found that the positive rate of mNGS for identifying fungal pathogens surpassed that of CMT in both immunocompromised and immunocompetent groups. Overall, mNGS shows strong utility in the detection of fungal infections. In fungal detection, mNGS significantly reduces the detection threshold by using mechanical homogenization or enzymatic cell wall rupture methods, thereby greatly improving analytical sensitivity (Zinter et al., 2019; Peng et al., 2021). This research found that the predominant fungal pathogens were *Candida albicans, Pneumocystis jirovecii, Aspergillus fumigatus, and Candida tropicalis.* It is important to emphasize that *Pneumocystis jirovecii*, which had the second highest detection rate, cannot be routinely cultured in clinical labs. This makes it easy to miss and delays the start of targeted treatment. The detection rate of *Aspergillus fumigatus*, which came in third, was greater with mNGS than with CMTs, which is in line with what previous investigations have shown (Jiang et al., 2021; Zhang et al., 2021; Chen et al., 2025). This illustrates that mNGS may identify hidden fungal infections, therefore furnishing doctors with more data to enhance accurate diagnosis and informed treatment decision-making.

In the ICU, fungal infections are mostly mixed infections, with just 14 cases classified as simple fungal infections; the majority are accompanied by concurrent bacterial and viral infections. Currently, the identification of viral infections in clinical practice mostly depends on PCR. Nonetheless, since PCR relies on pre-existing knowledge of target sequence information, its capacity to identify viruses is limited. Research indicates that when the pathogen is unidentified or the diagnosis is ambiguous, the viral detection rate of PCR is diminished. Our investigation revealed that the viral detection rate of mNGS was much superior to that of PCR, aligning with results documented in prior research (Chen et al., 2021b; Hao et al., 2023). The three predominant viruses linked to IFDs are *human gammaherpesvirus 4* (Epstein–Barr virus, EBV), *human betaherpesvirus 5* (cytomegalovirus, CMV), and *coronavirus*. Prior research indicates that *cytomegalovirus* and *herpes simplex virus* are susceptible to reactivation in ICU patients undergoing invasive mechanical ventilation, correlating with elevated death rates (Costa et al., 2012; Cowley et al., 2017). *Respiratory syncytial virus* is regularly identified in individuals with hematological disorders (Khanna et al., 2008), whereas *human parainfluenza virus type 3* is often seen in patients after hematopoietic stem cell transplantation (Kakiuchi et al., 2018). These findings suggest that different diseases may be accompanied by multiple distinct viral co-infections. The use of mNGS enhances viral detection capabilities, enabling physicians to adjust treatment approaches according to viral load and clinical presentations, thereby improving patient outcomes. A significant observation is that mNGS often identifies infections that are difficult to detect using CMTs. This investigation found pathogens including *Nocardia farcinica, Clostridium ramosum, Finegoldia magna, Exophiala dermatitidis*, and *Cryptococcus gattii.* Despite the rarity of these pathogens, co-infection with such organisms in ICU patients with pre-existing fungal infections significantly worsens disease severity and elevates mortality risk. Consequently, when patients exhibit inadequate responses to standard antimicrobial treatment and CMTs are constrained, clinicians can utilize mNGS to improve the detection rate of rare pathogens. In identifying this mixed infection, both mNGS and CMTs revealed mismatched and partly matched findings, irrespective of whether the subjects were in the immunocompetent or immunocompromised group. This phenomenon is not exclusive to fungal diseases; it is also seen in several other studies (Yang et al., 2024). This illustrates that mNGS can identify a wider array of pathogens. This advantage presents challenges for clinicians, as the interpretation of mNGS results necessitates caution and must be integrated with clinical manifestations and various contextual factors to differentiate normal colonizing microorganisms from genuine infectious pathogens, thereby enabling the formulation of an optimal therapeutic strategy.

The present study demonstrated that mNGS exhibits superior clinical value over CMTs in guiding anti-infective treatment and improving prognosis of critically ill patients, with varying performance characteristics between immunocompromised and immunocompetent subgroups. In the immunocompromised subgroup, mNGS significantly elevated the initiation rates of adjunctive antifungal and antiviral therapies and reduced ICU mortality compared with CMTs (Zhao et al., 2025). However, no significant difference was observed in antifungal regimen adjustment between the two groups. This may be explained by the unique immunosuppressive state of these patients, who are at extremely high risk of invasive fungal infections. Clinicians tend to implement empirical and prophylactic antifungal therapy based on clinical experience before definitive pathogen results are available. The fungal pathogens identified by mNGS are mostly consistent with the ongoing prophylactic antifungal treatment, leading to no further adjustment of antifungal regimens, thus resulting in a non-significant between-group difference (Garnacho-Montero et al., 2024). In contrast, for immunocompetent patients, mNGS guidance was associated with significantly higher rates of antifungal/antiviral therapy initiation and antifungal regimen adjustment, along with lower ICU mortality than CMTs, all with statistical significance. Unlike immunocompromised individuals, patients with normal immune function rarely require excessive empirical or prophylactic antifungal treatment without clear pathogen evidence. Therefore, accurate fungal detection results from mNGS can provide a reliable reference for targeted antifungal regimen optimization, showing greater guiding significance in this population. Overall, mNGS overcomes the limitations of CMTs such as low sensitivity and culture dependence, enabling rapid and precise pathogen identification. It effectively optimizes anti-infective treatment strategies and reduces ICU mortality in both immune subgroups. The non-significant difference in antifungal adjustment in immunocompromised patients reflects the combined effect of host immune status and clinical empirical prophylactic medication, highlighting the necessity of integrating pathogen detection with host characteristics for individualized anti-infective treatment (Li et al., 2023).

Our analysis revealed no significant difference in the percentage of clinically effective, mNGS-guided therapy between the immunocompromised and immunocompetent groups. However, patients may experience infections at multiple sites, and the choice of specimen types for testing has a direct impact on the effectiveness of pathogen identification diagnostics and treatment plan development. In some clinical situations, such as challenges in collecting samples from the infection site or the occurrence of fever of unclear cause, peripheral blood specimens may be selected for diagnostic assessment. In this study, the comparison of pathogen detection rates between mNGS and CMTs in blood specimens showed no significant difference, and treatment regimens remained largely unchanged, leading to the lowest observed clinical benefit. Our conclusion contrasts with several previously published studies (Chen et al., 2021a; Liu et al., 2023), which may be attributed to the fact that all fungal bloodstream infections in our investigation were caused by Candida species, known for their relatively high detection rate in blood cultures. The findings suggest that patients may not gain significant advantages from mNGS-based diagnostics when blood samples are submitted for fungus testing. Due to the prevalence of mechanical ventilation or endotracheal intubation among ICU patients, BALF is readily obtainable; consequently, BALF constituted the largest proportion of specimens in this study. In BALF, pus, urine, and drainage fluid specimens, mNGS results impacted treatment primarily by guiding adjustments in antimicrobial therapy. Its pathogen detection rate was much greater than that of CMTs, indicating that traditional approaches may offer insufficient coverage for certain specimen types, leaving some uncommon or difficult-to-culture infections unidentified. This discovery has been validated by other investigations (Chen et al., 2021b; Zhang et al., 2022; Hao et al., 2023). In sterile body fluids such as ascites and pleural effusion, mNGS results had the greatest clinical impact on treatment, with most cases leading to the initiation of targeted therapy. Consequently, it can be reasonably deduced that mNGS findings derived from specimens acquired in sterile conditions are more dependable than those from non-sterile situations. Multiple researches have also proven this trait (Wang et al., 2024; Wei et al., 2024; Zhan et al., 2024).

This research has several limitations. It was performed at a single center, and due to the limited patient population with fungal infections and the relatively small number of samples submitted for mNGS, the total sample size was constrained, potentially impacting the assessment of mNGS. Moreover, sterile specimens such as cerebrospinal fluid and joint fluid were not included, leading to incomplete coverage of specimen types. Furthermore, several specimen categories were characterized by minimal sample sizes, thus introducing bias in the interpretation of mNGS data. To enhance the evaluation of risk factors for fungal infections in immunocompetent individuals, further research should increase the sample size and use a multicenter approach.

In conclusion, this study yielded three notable findings. First, in the ICU, a substantial number of invasive fungal infections occurred even in patients without recognized risk factors. Second, mNGS demonstrated a broader pathogen spectrum and enhanced detection sensitivity in individuals with IFDs. Finally, the type of specimen provided for testing significantly impacted diagnostic efficacy and clinical benefit.

## Data Availability

The original contributions presented in the study are included in the article/**Supplementary Material**. Further inquiries can be directed to the corresponding author.
