# Supplementary material for: The complexity of invasive fungal diseases in the intensive care unit: evaluation of metagenomic next-generation sequencing
Source: Front Cell Infect Microbiol. 2026 May 29;16:1820501. doi: 10.3389/fcimb.2026.1820501 (PMC13261631; doi:10.3389/fcimb.2026.1820501)
Supplement: Supplementary file 1 [file DataSheet1.zip › Supplementary Material/Supplementary Material.docx]

Supplementary Material

Supplementary Materials: Impact of mNGS results on individual anti-infective therapy across different specimen types submitted for testing. (A) BALF, (B) blood, (C) Ascitic fluid, (D) Pleural effusion, (E) Others: urine, drainage fluid, pus.
