# Supplementary figures and images for: The complexity of invasive fungal diseases in the intensive care unit: evaluation of metagenomic next-generation sequencing
Source: Front Cell Infect Microbiol. 2026 May 29;16:1820501. doi: 10.3389/fcimb.2026.1820501 (PMC13261631; doi:10.3389/fcimb.2026.1820501)

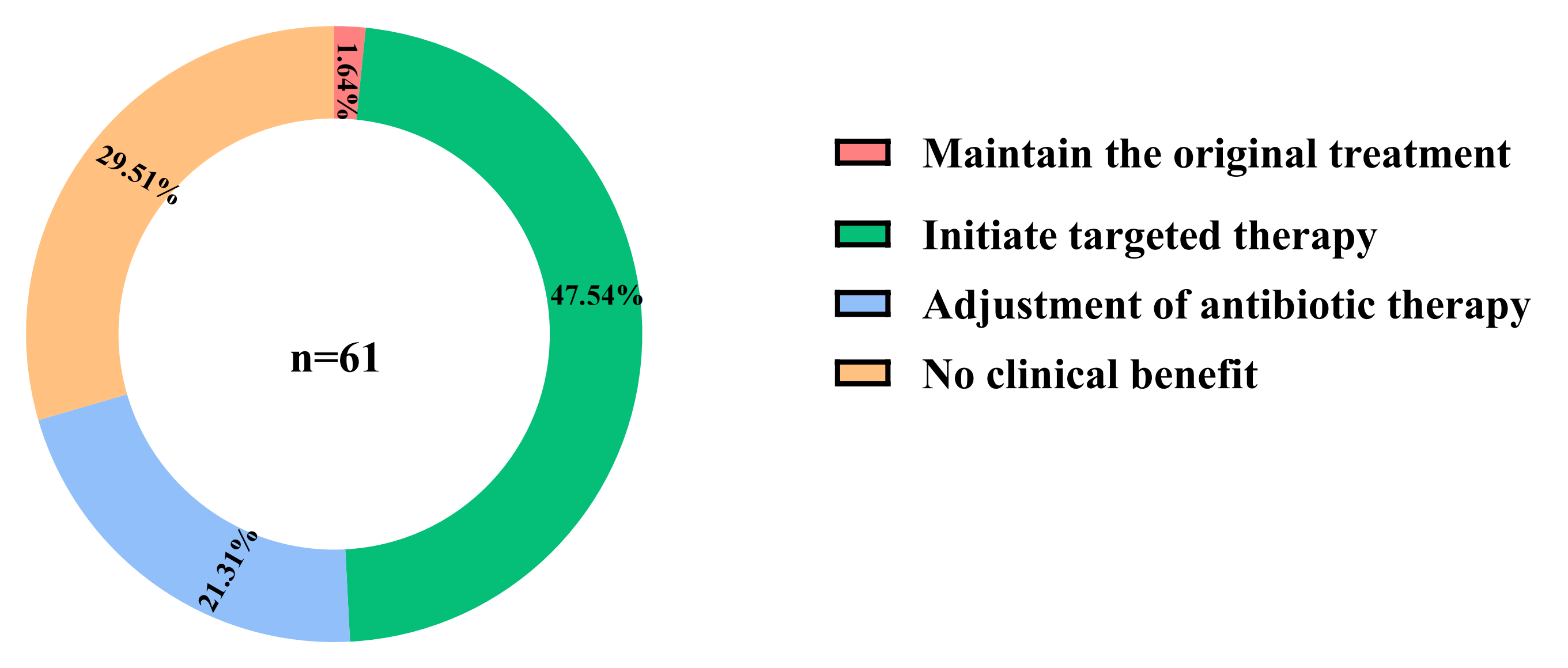

Supplement: Supplementary file 1 [file DataSheet1.zip › Supplementary Material/Figure A.tif]

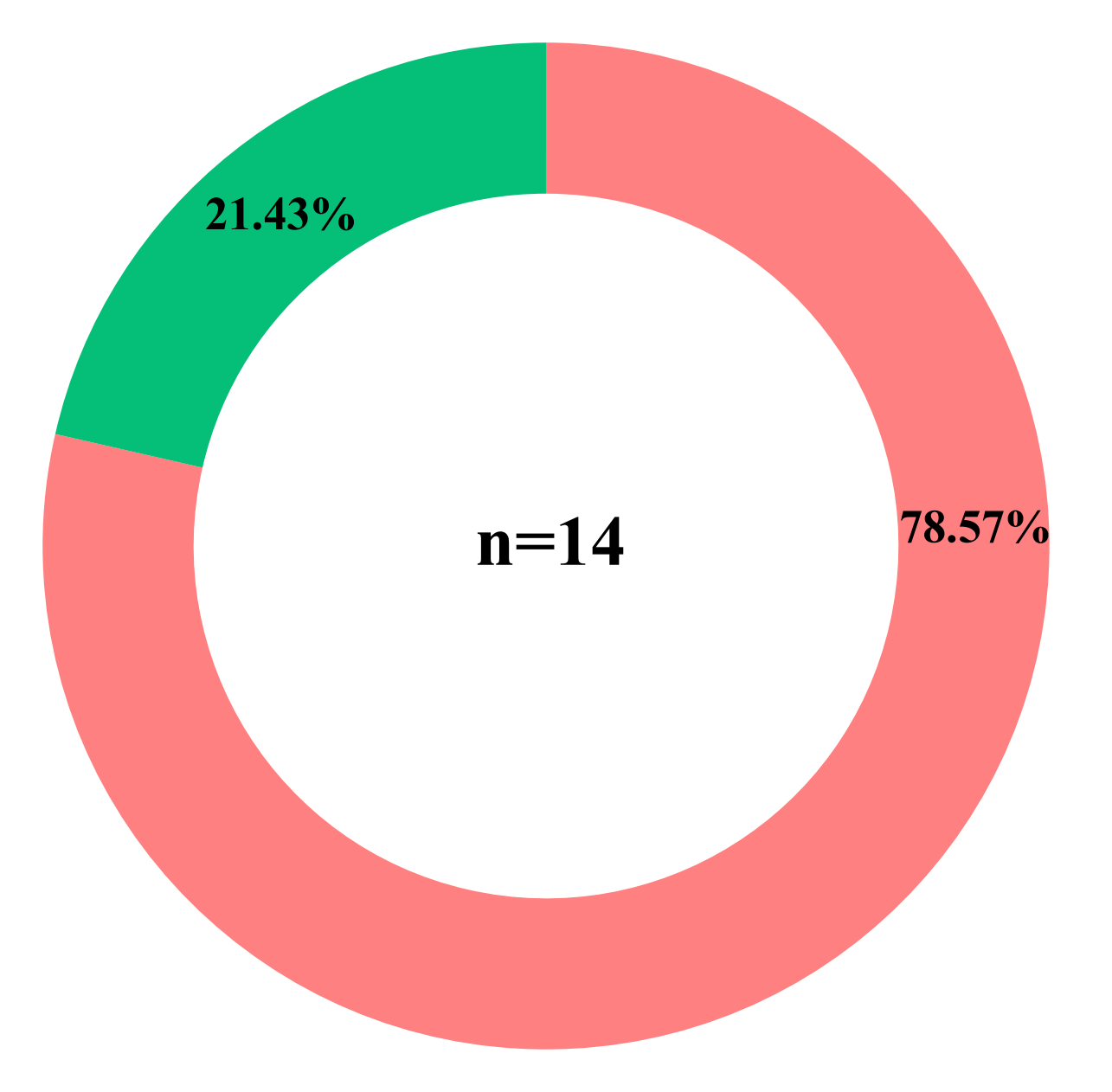

Supplement: Supplementary file 1 [file DataSheet1.zip › Supplementary Material/Figure B.tif]

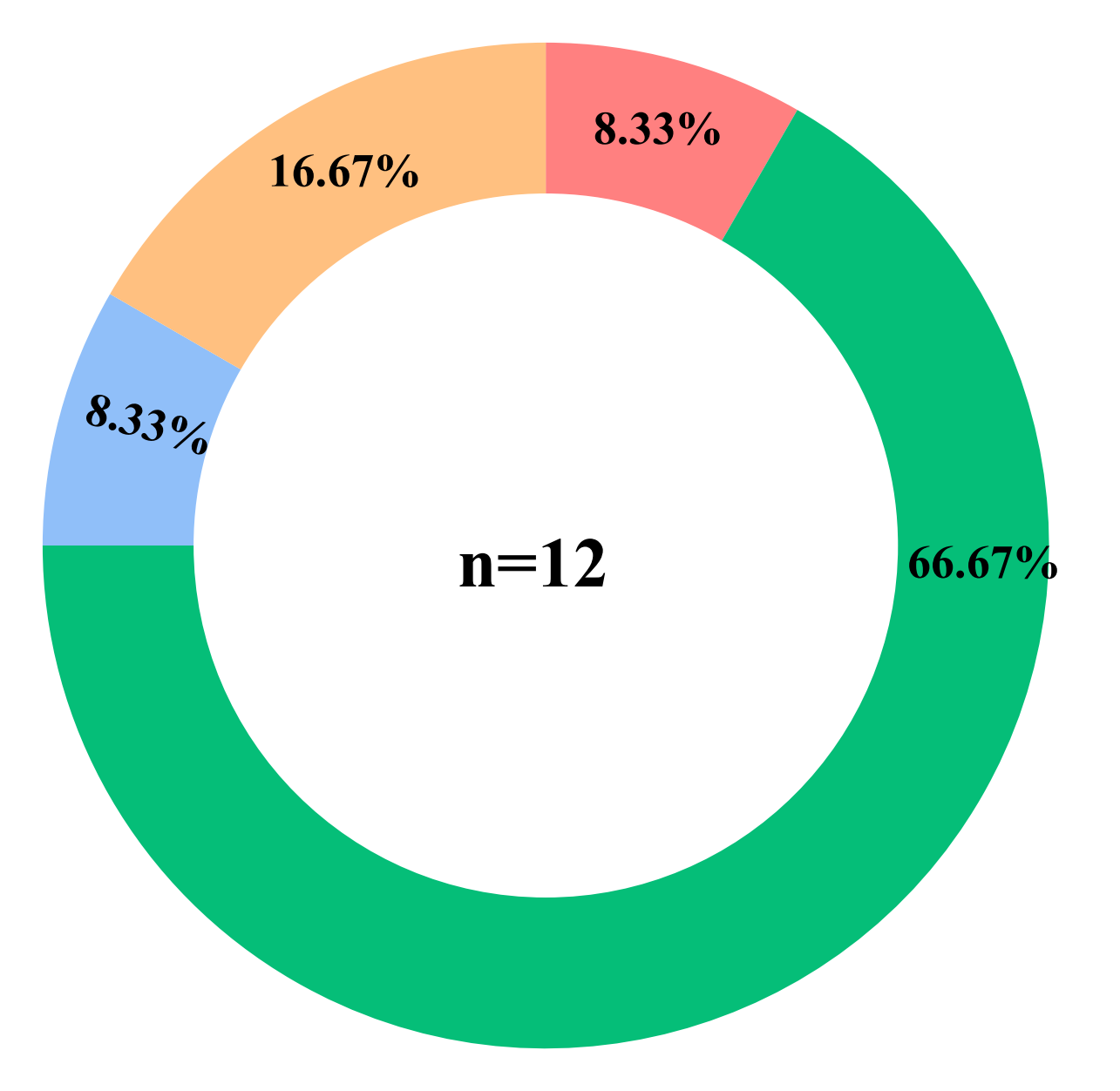

Supplement: Supplementary file 1 [file DataSheet1.zip › Supplementary Material/Figure C.tif]

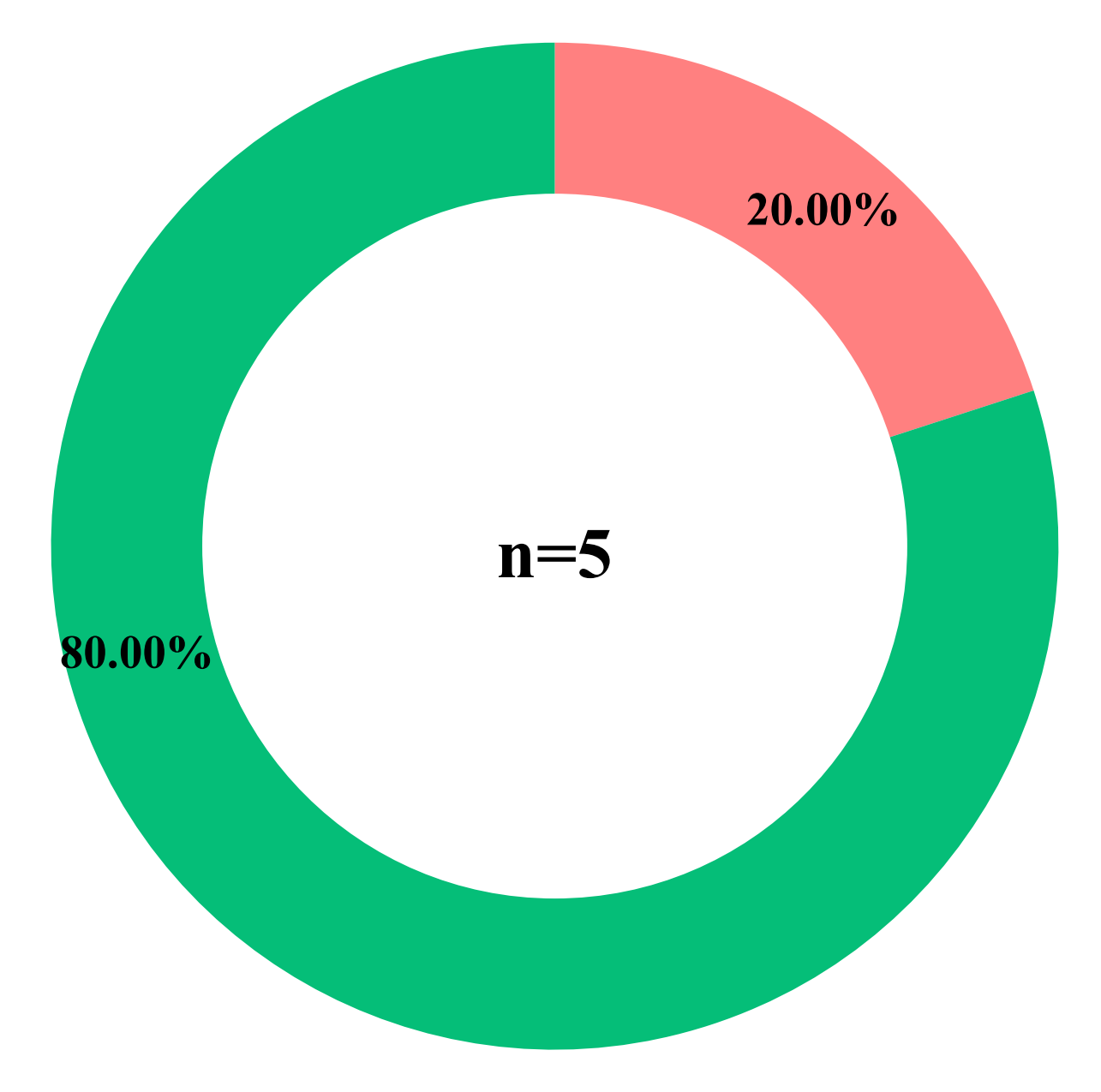

Supplement: Supplementary file 1 [file DataSheet1.zip › Supplementary Material/Figure D.tif]

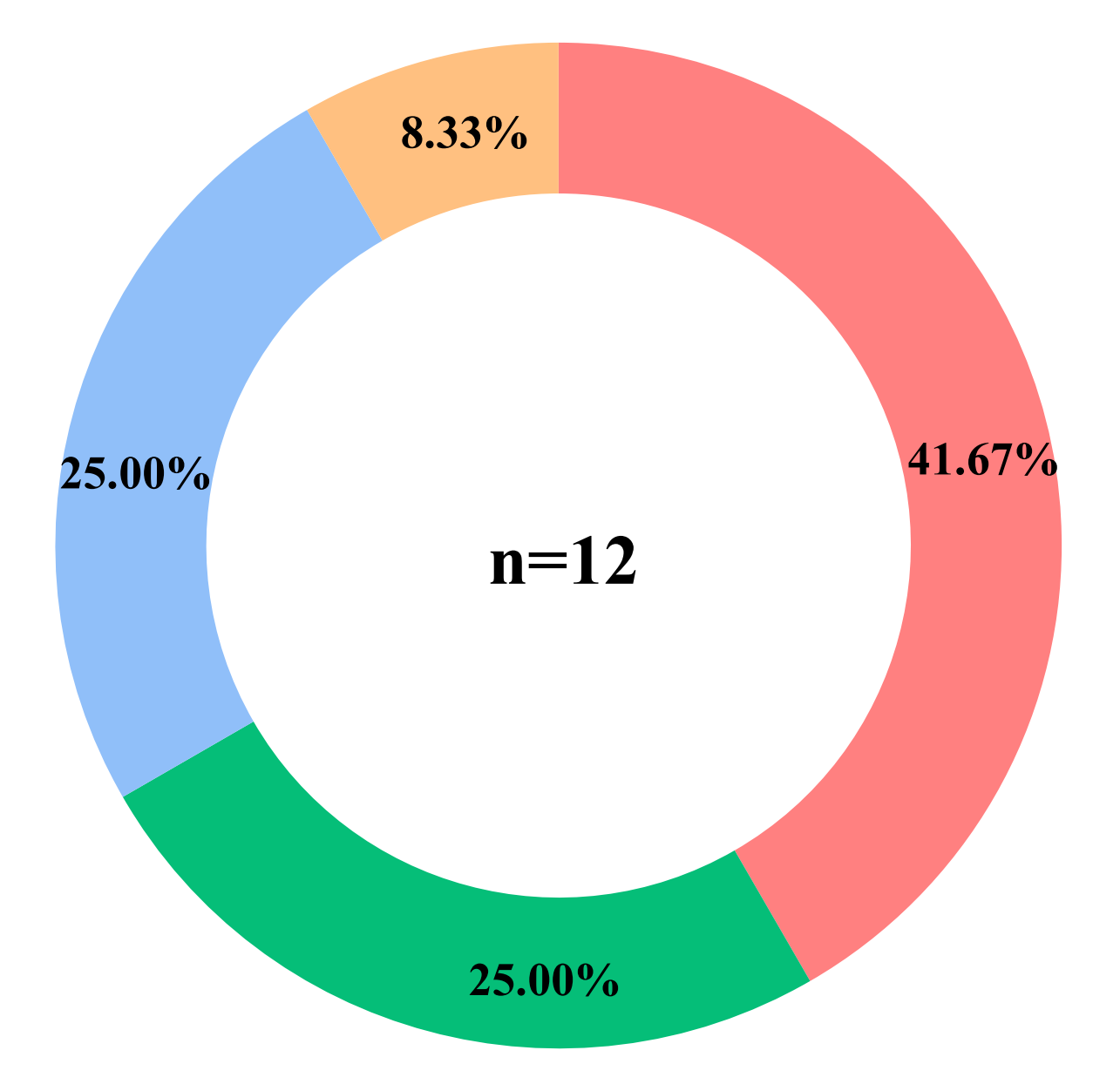

Supplement: Supplementary file 1 [file DataSheet1.zip › Supplementary Material/Figure E.tif]
